# Caffeine Exposure Alters Neurotransmission and Stress Physiology in a Freshwater Gastropod

**DOI:** 10.3390/toxics14050446

**Published:** 2026-05-20

**Authors:** Ahlam Mohamed-Benhamu

**Affiliations:** Grupo de Bioinformática y Ecotoxicología Molecular de Invertebrados, Facultad de Ciencias, Universidad Nacional de Educación a Distancia (UNED), Av. Esparta S/N, Las Rozas, 28232 Madrid, Spain; ahlam.mohamed@ccia.uned.es; Tel.: +34-91-398-7644

**Keywords:** water pollution, emerging pollutants, freshwater mollusks, gene expression, caffeine, nervous system, stress response

## Abstract

Caffeine (CAF) is a widely consumed psychostimulant known to modulate adenosine receptors and neurotransmitter systems, although its effects in invertebrates remain poorly understood. Environmentally relevant concentrations (5, 30, and 50 µg/L) are associated with altered behavior, including locomotion, exploration, and feeding, in the freshwater gastropod *Physella acuta*. This study examined molecular responses underlying these effects. Adult snails were exposed to CAF for 24 h and 7 days. Gene expression related to the nervous system and stress pathways was analyzed by RT-PCR, including *A1AR*, *ADORA2B*, *AChE*, *GLRA2*, *DRD2*, *RYR*, *HSD11β*, *HSP70*, *SLC6A2*, and *SLC6A1*. After 24 h, exposure to 50 µg/L CAF altered *A1AR* expression and caused downregulation of *AChE*, *GLRA2*, and *DRD2*, associated with observed behavioral changes. *A1AR* upregulation may indicate compensatory adjustment in adenosine signaling. After 7 days, *A1AR* remained upregulated, while genes linked to inhibitory neurotransmission showed partial recovery. Increased expression of genes involved in dopamine regulation and steroid metabolism suggested physiological adaptation. Overall, CAF induced dose- and time-dependent molecular responses in *P. acuta*, linking neurochemical disruption with behavioral changes and highlighting its ecological risk as an emerging freshwater contaminant.

## 1. Introduction

Caffeine (1,3,7-trimethylxanthine, CAF) is a purine alkaloid that acts as a potent stimulant on the central nervous system and is among the most widely consumed psychoactive substances found naturally in coffee, tea, and cocoa, as well as synthetically in pharmaceuticals, energy drinks, and personal care products (PCPs) [1]. Globally, daily consumption exceeds 460 tons [2].

Due to its widespread use, CAF has become ubiquitous in freshwater ecosystems, where it is commonly detected at varying concentrations depending on the water matrix [3,4,5]. In wastewater influents and effluents, reported concentrations vary substantially, from as low as 0.02 to as high as 86,000 µg/L. In surface waters such as rivers and lakes, concentrations typically range from 0.05 to 33.2 µg/L, while groundwater concentrations occasionally reach up to 0.68 µg/L. Drinking water shows levels from 0.50 to 35 µg/L, and reservoirs have recorded concentrations as high as 27.7 µg/L [6].

According to Nunes, B. et al. [4], CAF in wastewater commonly ranges from 20 to 300 µg/L, and some of the highest surface water concentrations have been reported in Costa Rica, reaching 1.1 mg/L [7], and in northwestern Spain, where levels reached up to 44.6 µg/L [8]. Although CAF is considered safe for human consumption at typical dietary levels, it has emerged as an emergent contaminant of concern in aquatic environments due to its potential effects on non-target organisms, particularly invertebrates [6].

This chemical exhibits remarkable stability under environmental conditions, such as fluctuations in salinity, light intensity, and temperature, which influence its degradation rates. In particular, photostability has been reported for up to three weeks under controlled conditions [9]. It is highly water-soluble (20 g/L) and persists in aquatic systems, with half-lives ranging from 100–240 days up to 10 years [6].

Ongoing inputs of human and industrial wastewaters often exceed their natural degradation rates, resulting in widespread detection not only in water but also in aquatic organisms, including fish species as *Gambusia holbrooki*, *Gerres oyena*, *Chanos chanos*, *Lethrinus nebulosus*, and *Oreochromis niloticus* [10]. Moreover, CAF can adsorb onto sediments and particulate matter, creating a secondary reservoir that prolongs its environmental persistence and allows gradual release back into the water column. Indeed, analysis of a 25 cm sediment core demonstrated that CAF was widely distributed throughout the profile, likely due to its high water solubility and mobility [11].

Despite its widespread environmental presence and long-term persistence, the biological effects of CAF, particularly in aquatic invertebrates, remain poorly understood. Current knowledge mechanisms are largely extrapolated from vertebrate models, such as rodents and *Danio rerio*, and from terrestrial invertebrates such as *Drosophila melanogaster*. In these organisms, CAF exposure can induce cellular stress responses, including heat-shock protein induction and alterations in metabolic enzymes, which may affect organismal health even at low environmental concentrations [6,12,13,14].

Moreover, in vertebrates, CAF-mediated effects are strongly linked to cyclic adenosine monophosphate (cAMP) signaling, calcium homeostasis, and adenosine receptor-mediated (A_1_ and A_2_A) pathways, which influence nervous system activity, metabolism, and stress responses [13,15,16]. Through these mechanisms, CAF modulates neurotransmission via adenosine receptor antagonism, indirectly influencing neurotransmitter release and affecting several signaling pathways, including cholinergic, dopaminergic, GABAergic, glutamatergic, and serotonergic systems [6,12,14,17]. The high affinity of CAF for adenosine receptors is considered central to its biological activity in mammals [17].

Evidence from terrestrial invertebrates indicates that CAF can also disrupt neurotransmission and cellular homeostasis. For example, CAF exposure increases cAMP concentrations in the brain of *Drosophila*, whereas direct application to the brain of honeybees elevates intracellular calcium levels [17]. In invertebrates, only interactions with ryanodine receptors (RYRs) and phosphodiesterase’s (PDEs) have been experimentally confirmed [17], highlighting potential differences in CAF’s molecular targets across taxa. However, comparable molecular-level studies in aquatic invertebrates remain limited, making it essential to elucidate how CAF affects these organisms to assess potential sub-lethal impacts and consequences for freshwater ecosystem health [6].

To address this knowledge gap, freshwater snails such as *Physella acuta* provide an ecologically relevant model to study CAF-induced molecular and physiological effects. This species is widely distributed in freshwater ecosystems, where it plays an important ecological role in periphyton grazing and nutrient cycling and is frequently used as a bioindicator of aquatic pollution. Its physiology, particularly its nervous and stress-response systems, makes it a suitable model for studying sub-lethal and molecular-level effects of environmental contaminants. Although CAF is widely detected in aquatic systems, its molecular effects on these organisms remain poorly characterized. It has previously been shown that *P. acuta* exhibits physiological and behavioral alterations when exposed to CAF at environmental levels [9]; however, the underlying molecular mechanisms have not yet been investigated. Given CAF’s known antagonist effects on adenosine receptors [17,18] and its broader modulation of neurotransmission and stress-related pathways, this study aimed to investigate the expression of genes associated with these biological systems in *P. acuta*.

Therefore, it was hypothesized that CAF, despite being considered a low-priority contaminant, can elicit significant biological alterations in non-target aquatic species. Accordingly, this study was designed to elucidate the molecular responses of *P. acuta* to environmentally relevant concentrations of CAF.

By linking these molecular markers to previously reported physiological and behavioral effects [9], this work suggests possible mechanistic connections to organism-level responses and could help inform a more comprehensive assessment of the ecological risks posed by CAF in freshwater environments.

## 2. Materials and Methods

### 2.1. Gene Characterization: Sequence Analysis and Domain Identification

**Figure 1:**
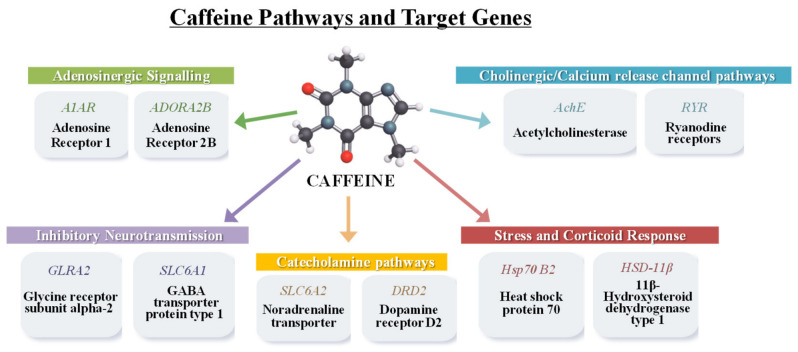
Schematic overview of proposed molecular and neurophysiological pathways affected by caffeine exposure. It is shown the different biological processes which have been described as sensitive to CAF: adenosinergic signaling, cholinergic release channel pathways, inhibitory neurotransmission, catecholamine pathways, stress and corticoid response.

Selected target genes were chosen based on their established roles in neurotransmission, stress regulation, and glucorticoid signaling, all of which are known or suspected targets of CAF action (Figure 1). Genes involved in adenosinergic signaling, including *adenosine receptor 1* (A1AR) and *adenosine A2B receptor* (ADORA2B), were analyzed, given CAF’s primary mechanism as an adenosine receptor antagonist [17,18]. Markers of cholinergic and dopaminergic neurotransmission, such as *acetylcholinesterase* (AChE) and *dopamine receptor D2* (DRD2), were included due to their roles in neurotoxicity and CAF-mediated modulation of motor and behavioral functions via adenosine–dopamine receptor interactions [18,19], while the *glycine receptor subunit alpha 2* (GLRA2) and *GABA transporter 1* (SLC6A1) were included to assess potential alterations in inhibitory neuro-transmission [17]. In addition, the *sodium-dependent noradrenaline transporter* (SLC6A2) and the *ryanodine receptor* (RYR) were selected as indicators of catecholaminergic signaling, stress responses, and calcium-mediated neuronal and muscular excitation [17]. To evaluate stress, *heat-shock protein 70* (HSP70 B2-like) was included as a molecular marker of cellular stress [20], and *hydroxysteroid 11β-dehydrogenase1* (HSD11β) for its potential involvement in CAF-induced modulation of stress responses and anxiety-like behaviors through neuroendocrine signaling [21].

These genes were initially identified by performing BLAST web server (NCBI) searches against a *Physella acuta* transcriptome to select homologous sequences corresponding to the proteins of interest (Table S1, Supplementary Material). Candidate sequences were validated against the NCBI database to confirm annotation, coding regions, and sequence identity. Only sequences with high similarity (low E-values), conserved functional regions, and complete or near-complete open reading frames (ORFs) were retained for further analyses.

**Figure 2:**
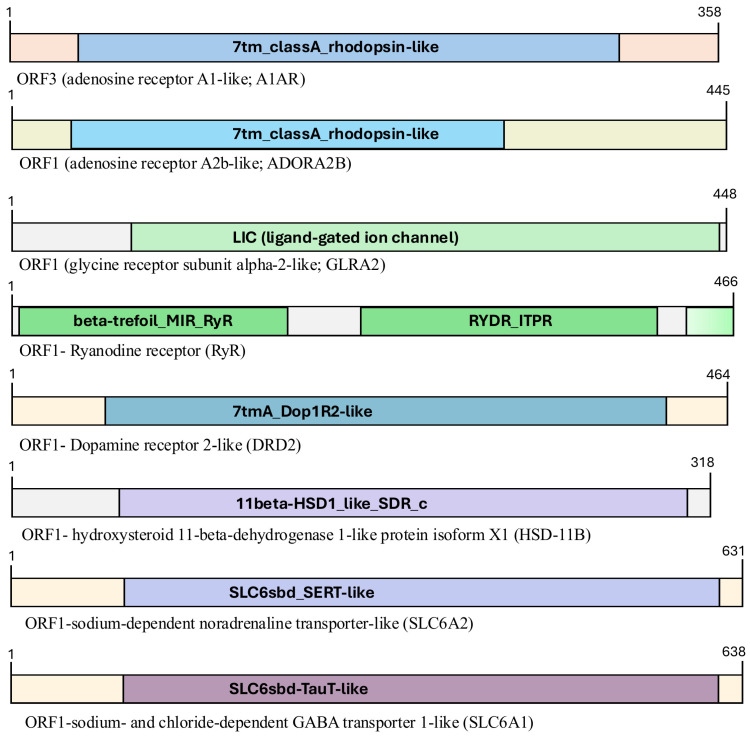
Structure and conserved domains of the identified *P. acuta* proteins. Each protein represents an open reading frame (ORF) derived from the sequences analyzed in this study. Proteins are schematically depicted, highlighting the conserved motifs and functional domains characteristic of each sequence. Domain architecture was predicted using the Conserved Domain Database (CDD). Protein sizes are indicated numerically.

The deduced protein sequences were analyzed using Conserved Domain Database (CDD) of the National Center for Biotechnology Information (NCBI) to identify conserved motifs, functional domains, and structural features. Graphical representations of the domain architecture and motif organization were generated using Microsoft PowerPoint (Version 16.108.3, Microsoft Corporation, Redmond, WA, USA), providing a clear visualization of key structural elements across the protein sequences (Figure 2). This workflow allowed comprehensive molecular characterization of the target genes and supported subsequent expression and functional studies.

### 2.2. Chemicals and Test Concentrations

Caffeine (C_8_H_10_N_4_O_2_, molecular weight 194.19 g/mol) was purchased from Sigma-Aldrich Chemical (St. Louis, MO, USA), with a degree of purity of 99%.

Nominal concentrations used were based on levels detected in environmental compartments, ranging from low nanograms to intermediate micrograms per liter [4], and have previously been shown to affect behavior [9]. Accordingly, three environmentally relevant caffeine concentrations were selected to expose the test organism, *P. acuta*: 5, 30, and 50 μg/L (25.75, 154.49, and 257.48 nM).

Caffeine stability under the same storage and experimental conditions was previously confirmed by nuclear magnetic resonance (NMR), showing no degradation over a one-month period [9].

### 2.3. Preparation Process of Concentrations Tested

Stock and exposure solutions were prepared according to the procedure described by [9]. Briefly, working solutions were obtained by direct dilution of the CAF stock solution (3 mg mL^−1^) in distilled water. The stock was stored at 4 °C and can be stored under refrigeration without light protection [9]. The high solubility and low hydrophobicity of CAF prevent adsorption onto glass surfaces, ensuring that measured concentrations accurately represent the aqueous medium.

### 2.4. Test Organisms and Experimental Setup

*Physella acuta* (Gastropoda, Pulmonata, Basommatophora) is a hermaphroditic species that mainly practices outcrossing. Before the experiment, it was maintained in a climate-controlled environment at 18 °C for several generations. The culture conditions were defined previously [22,23]. In summary, mature snails were kept in 0.75 L of culture medium (2 mM CaCl_2_, 0.5 mM MgSO_4_, 0.77 mM NaHCO_3_, and 0.08 mM KCl) at 18 °C under a 16:8 light–dark cycle for breeding purposes. Mature snails produced egg masses containing embryos, which developed directly into juveniles. As the juveniles matured into adults, their first oviposition occurred approximately two months after hatching.

To assess the effects of CAF exposure, short-term (24 h) and long-term (7 d) experimental conditions were established. For each exposure duration, a control group (non-exposed snails maintained under identical conditions without caffeine addition and non-solvent was used) and CAF-treated groups were included. Each treatment consisted of three independent experiments, with ten adult snails exposed per condition in glass vessels containing 100 mL of culture medium. Within each experiment, three snails per treatment were randomly selected for molecular analysis (*n* = 3), resulting in a total of nine biological replicates per treatment (*n* = 9) and an overall total of 72 samples.

This design was selected to ensure biological independence and capture inter-individual variability while minimizing pseudoreplication. Individual samples were analyzed separately (no pooling), allowing a more accurate assessment of variability in gene expression responses.

### 2.5. Behavioral Endpoints

Behavioral data, including feeding rate (as a proxy for appetite), exploration, speed, and trajectory parameters, were obtained from a previously published study [9]. In that work, behavioral endpoints were assessed under controlled laboratory conditions using standardized experimental protocols and video-tracking analysis. Briefly, organisms were recorded under defined exposure conditions, and behavioral metrics were extracted using automated tracking software. Full methodological details regarding experimental setup, recording conditions, and analytical procedures are described in [9].

### 2.6. RNA Isolation and Retrotranscription

Using TRIzol Reagent (Life Technologies, Carlsbad, CA, USA), total RNA was extracted following the manufacturer’s instructions. A final step was added using RNase-free DNase (Fisher, Madrid, Spain) for 45 min at 37 °C. RNase-free DNase was removed with a phenol:chloroform:isoamyl alcohol extraction (Fluka, Seelze, Germany). Isopropyl alcohol (0.5 *v*/*v*) was used to precipitate the total RNA, which was then washed with 75% ethanol and resuspended in 30 μL of DEPC water. RNA was quantified by absorbance spectrophotometry (Biophotomer Eppendorf, Hamburg, Germany). RNA samples were stored at −80 °C until reverse transcription (RT) was conducted. The RT reactions consisted of approximately 5 μg of RNA as template, 200 units of RevertAid Reverse Transcriptase (Thermo Fisher Scientific, Waltham, MA, USA), 0.5 μg of Oligo(dT)18 (Macrogen, Seoul, Republic of Korea), and 10 mM dNTPs (Biotools, Madrid, Spain). According to the manufacturer’s instructions, the reaction was made in a final volume of 40 μL at 42 °C for 60 min. The process was terminated by incubating at 70 °C for 10 min, and the cDNA was stored at −20 °C.

### 2.7. Real-Time PCR (qPCR)

Gene expression levels were analyzed using Real-Time PCR with primer pairs (Table 1), designed using Primer-BLAST (NCBI). The reaction was performed on a CFX96 thermocycler (Bio-Rad, Hercules, CA, USA) in a total volume of 10 µL, using 0.5 unit of DNA polymerase (Biotools, Madrid, Spain), 0.4 mM dNTPs, 2 mM MgCl_2_, and 0.5X EvaGreen (Biotium, Fremont, CA, USA). The RT-PCR conditions were described by [24].

The Real-Time PCR was run in the following cycling conditions: initial denaturation at 95 °C for 30 s followed by 39 cycles of 95 °C denaturation for 15 s, 58 °C annealing for 15 s, and 72 °C elongation for 30 s. A melt curve analysis was performed at the end of each run, increasing the temperature from 72 °C to 90 °C with incremental increases of 0.5 °C, to confirm amplification specificity and absence of primer-dimer formation.

*Glyceraldehyde 3-phosphate dehydrogenase* (GAPDH) and *Actin-β* (Act) genes were used as reference genes to normalize gene expression. Gene expression was analyzed using Bio-Rad Maestro software v.2.3, converting Ct values to relative expression levels via the 2^−ΔΔCt^ method [25]. A set of 10 unexposed adults, directly drawn from the cultures, was used as the external normalization condition for both control and treated samples. The mean Ct value for each gene was calculated from duplicate measurements and independent replicates. The efficiency of each primer set was determined using five 1:2 dilutions of a mixture of samples’ cDNA of equal molarity, performed in duplicate.

The stability of GAPDH and Act was evaluated by analyzing Ct variability across all experimental groups. Both genes showed comparable Ct dispersion with coefficients of variation below 10%, and ΔCt values between both genes remained relatively constant across samples, indicating no evidence of systematic regulation under experimental conditions. Although both reference genes individually showed acceptable stability, normalization was performed using the geometric mean of GAPDH and Act Ct values to improve robustness in heterogeneous biological conditions.

**Table 1:** Primer sequences described for the first time and PCR efficiencies for each gene. PCR efficiencies were calculated from standard curves. * Primer sequences for *AchE*, *Hsp70 B2-like*, *Actin* and *GAPDH* correspond to those reported by [26].

Gene	Primer	Sequence (5′ → 3′)	PCR Efficiency (%)
A1AR	F	CACGCATCTCGCTTGGAAGT	73.35
	R	AAGACAAAAGCACCGTCGCA	
GLRA2	F	GACGGCGAACTCTCAGCTTC	84.95
	R	AGGTGAAACTCGGCTTGCAG	
ADORA2B	F	AGCGAAGTAGTCGAGGCTGT	86.75
	R	CGACCTTTCGTCCGTCTGTG	
HSD-11B	F	CAGGACAAGACCCTGCAAGC	84.25
	R	CGTCCACGTCGTCCAGAAAG	
DRD2	F	GCAGCTCAAGCTCAGACAGA	83.30
	R	CGTGGCCTGATCCAAATCGT	
RYR	F	ACTGGCTGCCTTCTCAATCG	86.90
	R	TCATCACTTGGCTGCTCCAC	
SLC6A2	F	ATCGATAAGGTCGCCACGGA	90.02
	R	AGACCAAGGGAGAGCAGCAT	
SLC6A1	F	ATGGTGACGGAGGGTGGAAT	94.65
	R	ATGTCCCGCAGGTCATCGTA	
* HSP70 B2-like	F	CTGGAGGCGTTATGACTG	95.0
	R	AGGTGAAATCGACCCAAG	
* AchE	F	ATCAGTCGGGGCGAGATCAA	89.8
	R	AGTGCCGTTGAGAGGGAAGT	
* GAPDH	F	ATACATCAGGAACAGGGACTC	93.9
	R	GACTTATGACAACCGTGCA	
* Act	F	GAAGAGCTACGAGCTTCCCG	102.1
	R	CATGGATACCGGCAGACTCC	

### 2.8. Statistical Analysis

The mRNA levels of *A1AR*, *ADORA2B*, *AchE*, *GLRA2*, *DRD2*, *RYR*, *HSD11Β*, *SLC6A1*, *SLC6A2*, and *HSP70 B2-like* genes in response to CAF exposure were normalized against reference genes using the standard 2^−ΔΔCT^. All statistical analyses were performed in R (version 4.1.2) using the RStudio graphical interface (Posit Software, PBC, Version 2024.04.2+764) together with the packages *dplyr* and *tidyverse*. The normality of the data was assessed using the Shapiro–Wilk test. The Kruskal–Wallis test was applied to data that were not normally distributed. When significant differences were detected (*p* < 0.05), pairwise comparisons were performed using Dunn’s post hoc test. Bonferroni correction was applied within each gene across all pairwise treatment comparisons to control for multiple testing. No data transformations or outlier removal procedures were applied.

## 3. Results

**Figure 3:**
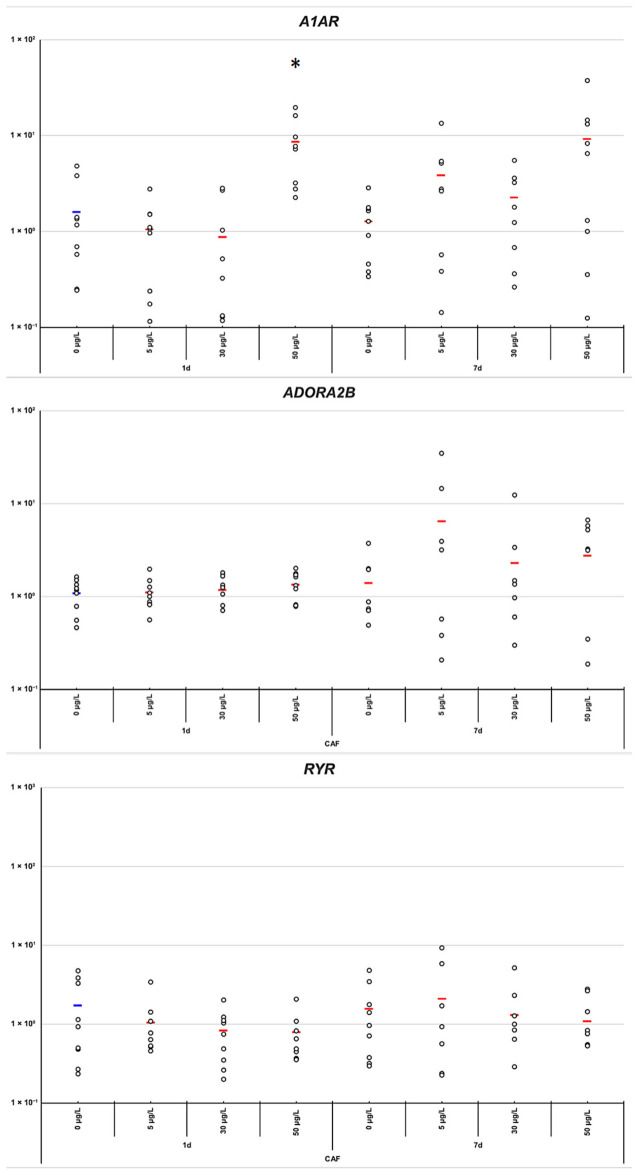
Gene expression levels of *ADORA2B*, *RYR*, and *A1AR* under varying concentrations of caffeine (0, 5, 30, 50 µg/L) and time points (1 and 7 days) of CAF treatment. Individual data points are shown (*n* = 9) with mean values in red. These genes are grouped as key components of the adenosine/Ca^2+^ signaling pathway, highlighting the coordinated expression changes observed in *ADORA2B* and *RYR* in response to treatment, while A1AR exhibits distinct regulation patterns. Asterisks (*) indicate statistically significant differences between control and treatment groups with *p*-values < 0.05.

**Figure 4:**
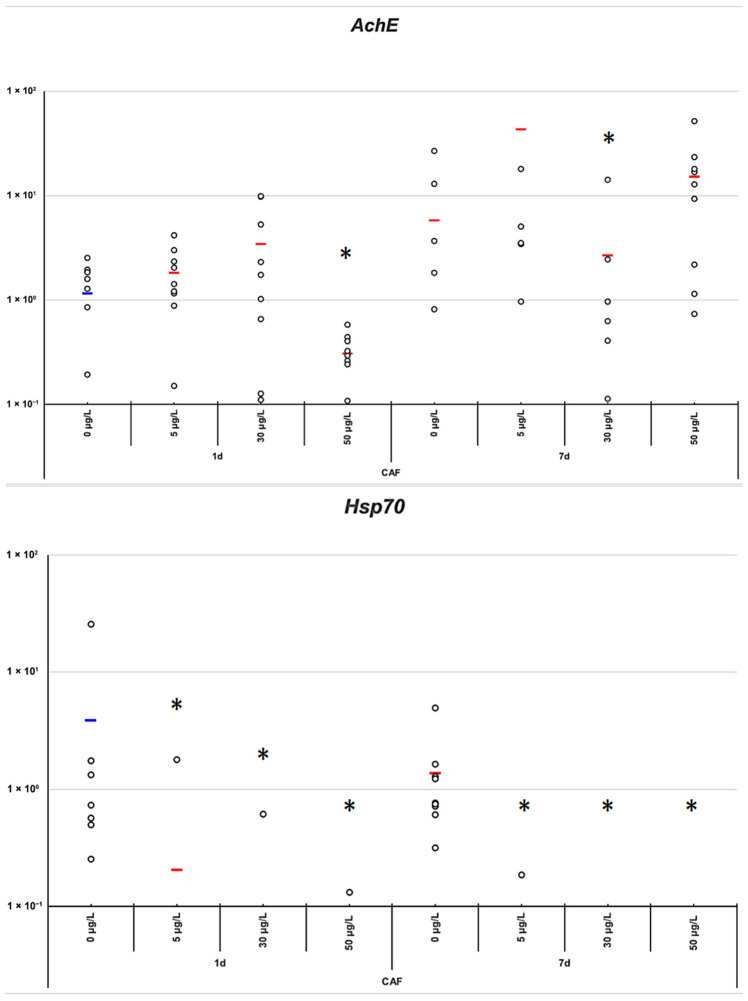
Gene expression levels of AChE and HSP70 B2-like under varying concentrations of caffeine (0, 5, 30, 50 µg/L) and time points (1 and 7 days). Individual data points are shown (*n* = 9) with mean values in red. These genes are selected as key caffeine-responsive markers. Asterisks (*) indicate statistically significant differences between control and treatment groups with *p*-values < 0.05.

**Figure 5:**
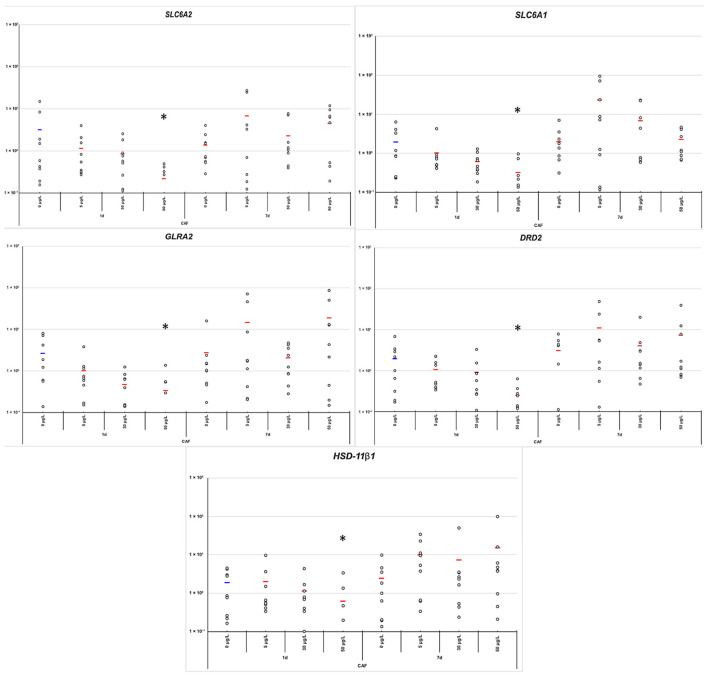
Gene expression levels of *SLC6A2*, *SLC6A1*, *GLRA2*, *DRD2* and *HSD11β*, under varying concentrations of caffeine (0, 5, 30, 50 µg/L) and time points (1 and 7 days). Individual data points are shown (*n* = 9) with mean values in red. These genes are selected as key components involved in neurotransmission and neuroendocrine signaling and exhibit a similar expression pattern, characterized by significant downregulation at 24 h following exposure to 50 µg/L caffeine and a compensatory response after 7 days of exposure. Asterisks (*) indicate statistically significant differences between control and treatment groups with *p*-values < 0.05.

CAF modulated the expression of several target genes, including *A1AR*, *AChE*, *GLRA2*, *DRD2*, *HSD11β*, *SLC6A2*, and *SLC6A1*. After 24 h of exposure, the 50 µg/L concentration induced a significant upregulation of *A1AR* (*p*-value = 0.0377) (Figure 3). In contrast, at this same concentration, a consistent downregulation is observed for *HSD11β*, *GLRA2*, *AChE*, *DRD2*, *SLC6A2* and *SLC6A1* (*p*-value = 0.0426, 0.0146, 0.0222, 0.0202, 0.0125, and 0.0285, respectively) (Figure 4 and Figure 5). No significant changes were observed in the expression of *ADORA2B* or *RYR* at any concentration and time point (Figure 3).

After 7 days of exposure, gene expression patterns shifted substantially. Genes initially downregulated at 24 h, including *HSD11β*, *GLRA2*, *DRD2*, *SLC6A2*, and *SLC6A1*, showed increased expression, particularly at 5 and 30 µg/L (Figure 5), returning to levels similar to those of the control.

Among the neurotransmission-related genes, *AChE* exhibited concentration- and time-specific responses. At 5 µg/L, *AChE* expression showed a tendency to increase at both 24 h and 7 days (Figure 4) (*p*-value = 0.0238 at 7 days), whereas at 30 µg/L, downregulation was observed at 7 days. Significantly, at 24h, concentration of 50 ug/L induced downregulation (Figure 4) (*p*-value = 0.0222). This pattern contrasts with other genes that exhibited a temporal inversion, suggesting that *AChE* may respond differently to CAF depending on exposure concentration and duration.

In addition to genes related to neurotransmission and metabolism, *HSP70 B2-like* displayed a distinct response profile. Across all concentrations and time points, CAF induced significant downregulation of *HSP70 B2-like* (Figure 4), which was more pronounced than the transcriptional changes observed for other genes (Kruskal–Wallis test, *p* = 1.68 × 10^−6^). This sharp downregulation was evident at both 24 h and 7 days, indicating that, unlike genes such as *HSD11β*, *GLRA2*, *AChE*, *DRD2*, *SLC6A2*, and *SLC6A1*, *HSP70 B2-like* did not exhibit compensatory activation with prolonged exposure. The consistency and magnitude of this response suggest that *HSP70 B2-like* may be one of the most sensitive molecular markers.

Consistent with the observed behavioral effects [9], low and medium doses were associated with increased locomotion and exploration at 24 h, matching early molecular changes, principally for *A1AR* and *AchE* (Figure 3 and Figure 4). By 7 days, this stimulation diminished alongside gene-expression shifts, consistent with adaptation. In contrast, high-dose exposure reduced movement at both time points, paralleling the strong early *A1AR* upregulation and *AchE*, *HSP70 B2-like* downregulation. Together, molecular and behavioral data suggest a dose- and time-dependent biphasic response to CAF.

In addition, the observed decrease in appetite at 24 h [9] coincided with a temporary downregulation of *DRD2* expression (control vs. 50 μg/L; *p*-value = 0.020). At 7 days, although appetite remained suppressed, *DRD2* expression recovered, suggesting a possible compensatory transcriptional response.

## 4. Discussion

Caffeine (CAF) modulated the mRNA expression of multiple target genes in *P. acuta*, including *A1AR*, *AChE*, *GLRA2*, *DRD2*, *HSD11β*, *SLC6A2*, and *SLC6A1*, eliciting apparent dose- and time-dependent responses. In contrast, no significant changes were detected in *RYR* expression at any concentration or time point, suggesting that mRNA levels of ryanodine receptor-mediated calcium signaling were not markedly affected under the experimental conditions tested.

Previous behavioral analyses in *P. acuta* reported CAF-induced biphasic changes in locomotion, exploration, and distance travelled, characterized by short-term stimulation at low concentrations (5 and 30 µg/L) and inhibition at higher doses (50 µg/L) or longer exposure times [9]. The present molecular results provide mechanistic support for these physiological responses, particularly through changes in adenosinergic, cholinergic, and glycinergic signaling pathways.

At low CAF concentrations, short-term stimulation of locomotion and overall behavior may be attributable to mild antagonism of adenosine receptors [18,27]. Adenosine normally exerts an inhibitory influence on locomotor activity; therefore, its blockade by CAF may reduce inhibitory tone, resulting in acute behavioral stimulation. Consistent with this, *A1AR* expression did not follow a simple dose-dependent trend but instead exhibited a pronounced time-dependent compensatory pattern. Short-term CAF exposure produced limited transcriptional changes at low concentrations, whereas significant upregulation of *A1AR* was observed at 50 µg/L after 24 h, indicating an early homeostatic response to strong receptor antagonism (Figure 3). By day 7, *A1AR* expression recovered and slightly exceeded control levels, consistent with sustained compensatory regulation of adenosinergic inhibitory signaling (Figure 3). This delayed receptor upregulation likely counteracts the acute behavioral stimulation induced by CAF and may contribute to the biphasic locomotor effects observed in *P. acuta* [9]. The concurrent late recovery of *GLRA2* further supports the presence of coordinated neuroadaptive mechanisms (Figure 5).

In addition to adenosinergic modulation, CAF exposure also affected genes associated with inhibitory glycinergic neurotransmission. *GLRA2* transcription exhibited a clear time-dependent pattern. After 24 h of exposure, *GLRA2* showed a tendency toward downregulation with increasing CAF concentrations, particularly at 5 and 30 µg/L, suggesting an acute reduction in glycinergic inhibitory signaling (Figure 5). Glycine receptors are key mediators of neuronal inhibition, and their reduced expression may contribute to increased neuronal excitability [28], which may be associated in turn with the short-term stimulation of locomotion and exploratory behavior observed at low CAF concentrations [9]. This interpretation is consistent with reports indicating that CAF acts as a weak competitive antagonist at ionotropic glycine receptors, functionally resembling strychnine at much lower potency [28].

By day 7, *GLRA2* mRNA levels were recovered at 5 and 50 µg/L (Figure 5), indicating a compensatory response aimed at restoring inhibitory tone following sustained or repeated receptor antagonism. This delayed upregulation coincides with increased *A1AR* transcriptional activity observed at later time points and is associated with adaptive neuroplastic mechanisms potentially related to the biphasic behavioral effects of CAF in *P. acuta* [9].

While adaptive changes were observed for *A1AR* and *GLRA2*, *HSP70 B2-like* was downregulated across all CAF concentrations (Figure 4). Unlike the receptors involved in inhibitory signaling, *HSP70 B2-like* did not show compensatory recovery, and its suppression did not always match with behavioral inhibition, which was evident only at high CAF doses. These results highlight *HSP70 B2-like* as a sensitive molecular marker of CAF-induced stress in *P. acuta*.

Alterations in *AChE* further support this interpretation. At 24 h, decreased *AChE* activity at the highest CAF concentration (50 µg/L) (Figure 4) coincided with locomotor suppression at this dose [9], whereas slightly increased *AChE* mRNA levels at 5 and 30 µg/L (Figure 4) may enhance cholinergic transmission, contributing to increased locomotor activity at lower doses. In combination with the changes in *A1AR* and *GLRA2*, these molecular responses could underlie the dose-dependent behavioral stimulation at low CAF concentrations and the suppression observed at higher concentrations.

CAF also modulated feeding behavior in a time-dependent manner. A previous study [9] reported that CAF may modulate feeding behavior in a dose-dependent manner; it was hypothesized that these effects may be mediated, at least in part, by dopaminergic signaling. Dopamine plays a central role in motivated behaviors, including feeding and locomotion, and disruption of dopaminergic pathways has been shown to reduce feeding drive across taxa [29,30]. In the present study, at 24 h, reduced feeding at 50 µg/L coincided with DRD2 downregulation in *P. acuta* (Figure 5), suggesting that early appetite suppression [9] may involve transient modulation of dopaminergic pathways.

By day 7, feeding remained suppressed despite *DRD2* recovery, indicating that compensatory molecular responses were insufficient to restore normal feeding behavior. *HSD11β*, *SLC6A1*, and *SLC6A2* exhibited similar temporal changes (Figure 5), supporting the interpretation that these genes are subject to coordinated homeostatic regulation under chronic CAF exposure. Persistent behavioral impairment despite transcriptional compensation suggests that additional mechanisms, such as altered presynaptic dopamine regulation, receptor sensitivity, or metabolic stress, may contribute to sustained feeding dysregulation.

This interpretation is consistent with previous reports indicating that feeding behaviors may be regulated by dopaminergic neural circuitry [30]. Moreover, the high degree of conservation of dopamine biosynthesis and transport pathways across species, including humans, *Drosophila* [31], and *C. elegans* [30], may support the potential relevance of these findings.

In addition to neurophysiological mechanisms, sensory factors may contribute to reduced feeding. CAF has a bitter and aversive taste, and decreased intake has been reported even at low concentrations, although the molecular pathways underlying this effect remain unclear [17]. Sensory avoidance may therefore act in parallel with neurochemical disruption to suppress feeding behavior under CAF exposure.

The similar dose- and time-dependent expression patterns observed for *DRD2* and *SLC6A1* suggest that CAF may elicit a coordinated homeostatic response within central neurotransmitter networks. Rather than reflecting opposing actions between dopaminergic and GABAergic pathways, these parallel changes likely reflect shared regulatory mechanisms that maintain neural stability under neurochemical stress. Such coordinated transcriptional adjustments could contribute to the modulation of behavioral state, without implying direct causal interactions between these genes. In parallel, *HSD11β* and *SLC6A2* exhibited comparable temporal dynamics, indicating broader homeostatic adaptations across monoaminergic and inhibitory pathways (Figure 5).

Although *DRD2*, *HSD11β*, *SLC6A1*, and *SLC6A2* exhibited similar temporal expression patterns (Figure 5), their functional roles in mediating CAF-related physiological effects are likely distinct. *DRD2* appears to be more directly involved in feeding-related motivation processes [32,33], whereas *HSD11β*, *SLC6A1*, and *SLC6A2* are involved in broader regulation of neurotransmission and behavioral state. Members of the *SLC6* transporter family regulate extracellular levels of key neurotransmitters, such as *GABA* and norepinephrine, and have been associated with behavioral phenotypes in previous studies [34,35,36], while *11β-HSD* enzymes modulate local glucocorticoid availability in neural tissues, influencing neural and stress-related behavioral responses [21].

Overall, CAF alters neurophysiological homeostasis in *P. acuta*, primarily through adenosinergic and dopaminergic pathways. Changes in *AChE* mRNA levels and *A1AR* activity suggest disruptions in cholinergic and purinergic signaling, particularly at high doses, resembling effects reported in vertebrates and other invertebrates [17]. CAF also induces coordinated, temporally structured molecular responses across inhibitory (*SLC6A1/GLRA2*) and monoaminergic (*DRD2/SLC6A2*) pathways, with *HSD11β* and *HSP70 B2-like* potentially serving as markers of metabolic or stress-related adaptations.

However, some genes showed limited or unclear responses, such as *RYR* and *ADORA2B*, highlighting important knowledge gaps. Additionally, information regarding the absorption, tissue distribution, and metabolites of CAF in invertebrates represents a significant gap in our understanding [17].

This study identifies consistent changes in gene expression following CAF exposure that were observed alongside physiological and behavioral responses, providing an initial step toward understanding its effects in freshwater invertebrates. While integrating these responses within an Adverse Outcome Pathway (AOP) framework would help link molecular changes to higher-level outcomes, this lies beyond the scope of the present work. Overall, the findings emphasize the need for further research to clarify the mechanisms through which CAF influences neurotransmission and behavioral regulation.

## 5. Conclusions

CAF exerts dose- and time-dependent effects in *P. acuta*, with low concentrations transiently stimulating locomotion, exploration, and feeding, whereas high concentrations lead to behavioral suppression and sustained transcriptional changes in *A1AR*, *GLRA2*, *DRD2*, *SLC6A1/SLC6A2*, *HSD11β*, and *HSP70 B2-like*. These results suggest that CAF may modulate multiple neurotransmission and stress-response pathways in a temporally structured manner, producing biphasic behavioral effects and indicating potential neuroadaptive mechanisms. Overall, the findings provide preliminary insights into the molecular and behavioral impacts of CAF in freshwater mollusks and emphasize the importance of exposure dose, duration, and further mechanistic studies in invertebrate neurophysiology.

## Data Availability

The original contributions presented in this study are included in the article/Supplementary Material. Further inquiries can be directed to the corresponding author.

## Supplementary Materials

The following supporting information can be downloaded at: https://www.mdpi.com/article/10.3390/toxics14050446/s1, Table S1: Predicted and characterized genes identified in the study, along with their GenBank accession numbers.

## Abbreviations

The following abbreviations are used in this manuscript:

CAF	Caffeine
NMR	Nuclear magnetic resonance
