# Supplementary material for: Caffeine Exposure Alters Neurotransmission and Stress Physiology in a Freshwater Gastropod
Source: Toxics. 2026 May 20;14(5):446. doi: 10.3390/toxics14050446 (PMC13211357; doi:10.3390/toxics14050446)
Supplement: Supplementary file 1 [file toxics-14-00446-s001.zip › toxics-4293607-supplementary.pdf]

Supplementary Material

Caffeine Exposure Alters Neurotransmission and Stress Physiology in a Freshwater Gastropod

Ahlam Mohamed-Benhamu

Grupo de Bioinformática y Ecotoxicología Molecular de Invertebrados, Facultad de Ciencias, Universidad Nacional de Educación a Distancia, UNED, Av. Esparta S/N, Las Rozas, 28232 Madrid, Spain; ahlam.mohamed@ccia.uned.es; Tel.: +34-91-398-7644

Table S1: Predicted and characterized genes identified in the study, along with their GenBank accession numbers.

Gene	Accession number
**PREDICTED - glycine receptor subunit alpha-2-like**	XM_059284123
**PREDICTED - adenosine receptor A1-like**	XM_059285151
**PREDICTED - adenosine receptor A2b-like**	XM_059286610
**PREDICTED - hydroxysteroid 11-beta-dehydrogenase 1-like protein isoform X2**	XM_059302598
**PREDICTED - dopamine receptor 2-like**	XM_059307500
**PREDICTED - ryanodine receptor-like**	XM_059306242
**PREDICTED: Sodium- and chloride-dependent GABA transporter 1-like (LOC131931958)**	XM_059288800
**PREDICTED: Sodium-dependent NORADRENALINE transporter-like (LOC131930796)**	XM_059287421
**Acetylcholinesterase**	MW456922
**Heat shock protein 70 B2-like**	OK474812
